# Glomerulonephritis and Interstitial Nephritis Originating from Vasculitis of the Interlobular Arteries of the Kidney in a Patient with Eosinophilic Granulomatosis with Polyangiitis

**DOI:** 10.1155/2022/9606981

**Published:** 2022-09-28

**Authors:** Takashi Nawata, Masaki Shibuya, Yukio Takeshita, Makoto Kubo, Noriko Uesugi, Masafumi Yano

**Affiliations:** ^1^Department of Medicine and Clinical Science, Yamaguchi University Graduate School of Medicine, Ube, Japan; ^2^Department of Neurology and Clinical Neuroscience, Yamaguchi University Graduate School of Medicine, Ube, Japan; ^3^Department of Pathology, Faculty of Medicine Fukuoka University, Fukuoka, Japan

## Abstract

Eosinophilic granulomatosis with polyangiitis (EGPA) is a type of antineutrophil cytoplasmic antibody-associated vasculitis. Patients often present with peripheral neuropathy and purpura, suggesting impairment of small vessels, especially capillaries. However, medium-sized vessels and small vessels with a vascular diameter larger than that of capillaries may also be impaired, causing atypical findings. We report a case of EGPA treated with corticosteroids, cyclophosphamide, and mepolizumab. Renal biopsy revealed vasculitis of the interlobular arteries as the cause of glomerulonephritis and interstitial nephritis. This case suggests the importance of considering vessels upstream of capillaries dominant EGPA as a differential diagnosis in patients with eosinophilia.

## 1. Introduction

Eosinophilic granulomatosis with polyangiitis (EGPA) is a type of anti-neutrophil cytoplasmic antibody (ANCA)-associated vasculitis (AAV), which affects small and medium-sized vessels [1]. EGPA is histologically characterised by fibrinoid necrosis, granuloma formation, and vessel inflammation caused by eosinophils [2]. This leads to the dysfunction of various organs in the form of peripheral neuropathy, skin lesions, and lung infiltrates [2, 3]. Renal involvement, such as via pauci-immune necrotizing glomerulonephritis, also occurs in approximately 20% of patients with EGPA [3]. The involvement of these organs, which have abundant small vessels such as capillaries, ordinally suggests the impairment of small vessels [4]. Here, we report a case of EGPA accompanied by glomerulonephritis and interstitial nephritis. Renal biopsy results showed that vasculitis of the interlobular arteries, which are small vessels with a larger vascular diameter than that of capillaries, was the major cause of glomerulonephritis and interstitial nephritis.

## 2. Case Presentation

A 77-year-old Japanese man was admitted to our hospital for fever. Ten years before admission, the patient had been diagnosed with adult-onset bronchial asthma, which was well controlled with inhaled steroid therapy. One month before admission, the patient developed a fever. When he visited a local hospital, a blood test revealed eosinophilia. Based on this result, he was suspected of having an autoimmune disease and was admitted to our hospital.

On admission, the patient's vital signs were as follows: blood pressure, 105/70 mmHg; pulse rate, 88 bpm; body temperature, 37.5°C; respiratory rate, 12 breaths/min; and SpO2, 97% in ambient air. There were no specific physical findings suggestive of systemic vasculitis, such as purpura and livedo reticularis. In addition, a physical examination did not reveal any abnormal findings suggesting other systemic diseases. A neurological examination did not indicate neuropathy.

A complete blood cell count showed mild normocytic anaemia and elevated leukocytes and platelets (haemoglobin, 10.7 g/dL; leukocytes, 16,790/µL; and platelets, 49.3 × 104/*μ*L). The differential leukocyte count showed that the number of eosinophils was elevated at 4,617/*μ*L. Laboratory investigations revealed elevated serum levels of C-reactive protein and erythrocyte sedimentation rate (12.7 mg/dL and 95 mm/h, respectively). Cytoplasmic ANCA (c-ANCA) detected via indirect immunofluorescence was positive. In contrast, perinuclear ANCA (p-ANCA) detected via indirect immunofluorescence and antiglomerular basement membrane antibodies detected by fluorescence enzyme immunoassay were negative. Antinuclear antibodies and rheumatoid factors were also negative. The serum IgG level was within normal limits (1,370 mg/dL), but IgE levels were elevated (229 IU/mL; normal range: <139 IU/mL). Although the serum levels of creatinine were normal (0.75 mg/dL; normal range: <1.07 mg/dL), the serum cystatin C level was elevated (1.39 mg/L; normal range: <0.95 mg/L), which suggested renal dysfunction. The protein-to-creatinine ratio in the spot urine sample was 0.22 g/gCr. Urine sediment analysis revealed abnormal urinary casts, including red blood cell casts, white blood cell casts, fatty casts, granular casts, and oval fat bodies. Urinary levels of N-acetyl-*β*-D-glucosaminidase and *β*2-microglobulin were also elevated (64.7 IU/L, normal range: <11.2 IU/L; and 0.34 mg/L, normal range: <0.15 mg/L, respectively). Based on the results of the urine tests and urine sediment analysis, glomerulonephritis and interstitial nephritis were suspected. A whole-body computed tomography (CT) scan revealed mild mucosal thickening of the right maxillary sinus and bilateral ethmoidal sinus and a lack of abnormal shadowing of the lung fields. A gastrointestinal endoscopy did not reveal any bleeding, while echocardiography did not show pericardial effusion. Lastly, a nerve conduction study showed no abnormal findings.

**Figure 1:**
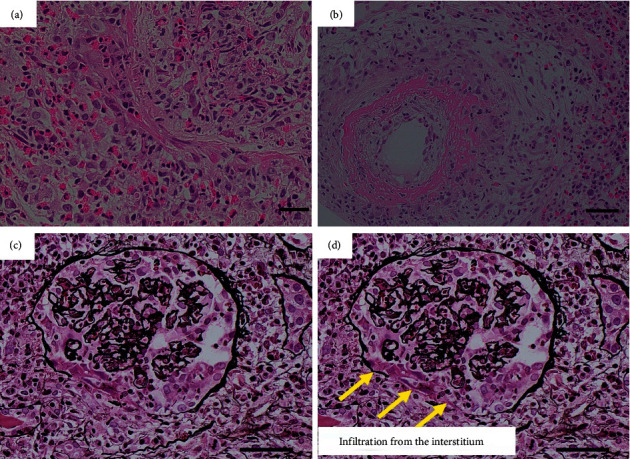
(a) Renal biopsy showing remarkable infiltration of eosinophils in the interstitium of the kidney (scale bar: 100 *μ*m). (b) Fibrinoid necrosis visible in the interlobular arteries of the kidney (scale bar: 50 *μ*m). (c) One glomerulus, showing glomerular crescent formation (scale bar: 50 *μ*m). (d) Rupture of the glomerular basement membrane in this glomerulus, which appears to have spread from outside of the glomerulus; the yellow arrows indicate infiltration from the interstitium (scale bar: 50 *μ*m). A B: haematoxylin and eosin staining, C D: periodic acid methenamine silver staining).

Renal biopsy revealed a remarkable infiltration of eosinophils in the interstitium of the kidney. In addition, fibrinoid necrosis was observed in the interlobular arteries, which have a relatively large vascular diameter in small vessels. A granuloma formation near the necrotic vascular lesion was also found. Almost all glomeruli in the biopsy specimen did not show any morphological abnormalities. However, only one glomerulus showed glomerular crescent formation. Interestingly, rupture of the glomerular basement membrane in this glomerulus appeared to have spread from outside of the glomerulus. These histopathological findings suggested that glomerulonephritis and interstitial nephritis had developed from the spread of inflammation originating from the interlobular arteries of the kidney (Figure 1).

According to the ACR 1990 classification criteria for EGPA [5], the patient was classified as having EGPA. There were no obvious findings to suggest other autoimmune diseases, such as polyarteritis nodosa, rheumatoid vasculitis, and Behcet's disease, which could indicate medium-size-vessel vasculitis. He was diagnosed as having EGPA and was treated with a 3-day course of steroid pulse therapy (methylprednisolone, 1 g/day) followed by oral prednisolone (PSL, initial dose, 50 mg/day, 1 mg/kg/day) along with intravenous cyclophosphamide (IVCY, 500 mg/body). The fever disappeared after initiating immunosuppressive therapy. Eosinophilia and abnormal urinalysis results also improved. The patient was scheduled to undergo six courses of IVCY. However, he developed cytomegalovirus infection and bone marrow suppression due to IVCY and valganciclovir, making the continuous administration of IVCY difficult. Therefore, cyclophosphamide was changed to subcutaneous mepolizumab (300 mg/month) during the treatment. After a gradual tapering of PSL, the patient was discharged home, where he received regular outpatient treatment.

## 3. Discussion

A history of adult-onset bronchial asthma and acute eosinophilia accompanied by various systemic symptoms is characteristic of EGPA [2]. Peripheral neuropathy and skin lesions are known to be the more frequent symptoms of EGPA [2]. In contrast, renal involvement in EGPA is less common than in other types of AAV, such as microscopic polyangiitis and granulomatosis with polyangiitis [3]. Therefore, there are few reported cases in which nephropathy due to EGPA causes clinical problems, and as such, nephropathy is not considered a prominent manifestation of EGPA [6].

Nasal mucosa biopsy has a low diagnostic value and sensitivity, with less than 10% demonstrating eosinophilic granuloma or vasculitis [7]. In our case, mucosal thickening of the right maxillary sinus and bilateral ethmoidal sinus were mild. Therefore, it was difficult to obtain pathological findings by nasal mucosa biopsy. Thus, in the present case, the obvious organ symptoms associated with EGPA were due to renal involvement only, which initially made it difficult to consider the possibility of EGPA. If a renal biopsy is not performed, similar patients may receive mild immunosuppressive treatment for idiopathic hypereosinophilic syndrome [8], which might not be able to suppress the disease activity completely, leading to life-threatening outcomes.

AAV is often assumed to affect only small vessels, especially capillaries. However, our case highlights two important aspects. First, small vessels with a vascular diameter larger than that of capillaries are sometimes impaired in AAV, including EGPA [2]. When medium-sized vessels or small vessels with a larger vascular diameter than capillaries are predominantly impaired, atypical symptoms of EGPA may occur [9,10]. Clinicians should thus keep in mind the possible impairment of vessels upstream of capillaries when presented with patients with eosinophilia accompanied by atypical findings of EGPA. Second, our case also suggests the importance of closely examining urinary and renal biopsy findings. In our case, proteinuria was barely noticeable while the abnormal urine sediment analysis results yielded the primary evidence of renal involvement, which was the indicator that highlighted the need for a renal biopsy.

It has been reported that pure acute interstitial nephritis is a rare renal presentation of EGPA [11]. However, more aggressive renal biopsy implementation in clinical practice may reveal pathological lesions similar to those in our case. Further case reports and studies on medium-sized vessel-associated symptoms and renal involvement of EGPA are required.

## Data Availability

Considering the patient privacy as an ethical concern, access to data is restricted.
